# 865. Fresh Frozen Plasma May Decrease Levels of Endothelial Dysfunction in Burn Injury Patients

**DOI:** 10.1093/jbcr/irag033.335

**Published:** 2026-04-06

**Authors:** Anna Mermilliod, Cara D Ramos, Dhanushka Vitharana, Abdul-Razak Masoud, Kaitlyn Andre, Claudia Leonardi, M Victoria P Miles, Jeffrey E Carter, Jonathan E Schoen, Alison A Smith

**Affiliations:** Louisiana State University Health Sciences Center, River Ridge, LA; Louisiana State University Health Sciences Center, New Orleans, LA; University Medical Center Burn Center, Louisiana State University Health Sciences Center, New Orleans, LA; Louisiana State University Health Sciences Center, New Orleans, LA; Louisiana State University Health Sciences Center, New Orleans, LA; Louisiana State University Health Sciences Center, New Orleans, LA; University Medical Center Burn Center, Louisiana State University Health Sciences Center, Department of Surgery, New Orleans, New Orleans, LA; University Medical Center Burn Center, Louisiana State University Health Sciences Center, New Orleans, LA; UCI Health Regional Burn Center, New Orleans, LA; University Medical Center Burn Center, Louisiana State University Health Sciences Center, New Orleans, LA

## Abstract

**Introduction:**

Patients with major burn injuries are resuscitated with a combination of crystalloids and colloids. Fresh frozen plasma (FFP) can also be given to patients with high total body surface area (TBSA) burns as an adjunctive colloid solution during burn resuscitation. FFP may reduce the endothelial dysfunction associated with large TBSA burns. Further, FFP may play a role in changing the patient’s inflammatory state by affecting cytokine levels released by adipose-derived stem cells (ADSCs), specifically the cytokine, VEGF-A. This study aimed to investigate a beneficial role of FFP on VEGF-A levels in burn patients.

**Methods:**

Following IRB approval, adipose tissue was collected from adult patients with full thickness burn injury during a post-injury surgery, with a median of four days following burn injury. ADSCs were isolated from the tissue by trypsin enzyme digestion. Fluorescence activated single cell sorting (FACS) of ADSCs was performed to determine purity with CD105, CD90, and CD73 antibodies. ADSCs were grown under standard tissue culture conditions and the supernatant was collected for cytokine analysis. Data were analyzed using a Spearman correlation of VEGF-A levels and amount of FFP received.

**Results:**

Fourteen patients receiving FFP during the first 36 hours post-burn injury were enrolled in this study, along with three patients that did not receive any FFP. The amount of FFP given to the 14 patients ranged from 258-3186 mL, with an average of 1465□715 mL. Average TBSA for all patients was found to be 35□24% and an average patient age of 56□17 years. Though statistical analysis demonstrated a moderately strong negative correlation between VEGF-A and FFP (Spearman correlation coefficient was measured at -0.458), this correlation was not statistically significant (*p*=.064).

**Conclusions:**

Though statistical analysis demonstrated a moderately strong negative correlation between VEGF-A and FFP, this correlation was not statistically significant. VEGF-A has previously been shown to have a role in angiogenesis, which can increase inflammatory cell infiltration and lead to endothelial cell dysfunction. Patients who received higher FFP levels are correlated with lower levels of VEGF-A, indicating there may be a correlation between higher dosage of FFP and decreased endothelial cell dysfunction. Future studies are required to increase sample size to further investigate this finding.

**Applicability of Research to Practice:**

This research can be used in practice to inform burn resuscitation protocols to guide more targeted use of FFP for major burn injuries.

**Funding for the study:**

This research was partially funded by the 2022 American Association for the Society of Trauma Research and Education Fund Trauma Critical Care Scholarship.

